# Comparative genomics of *Fervidobacterium*: a new phylogenomic landscape of these wide-spread thermophilic anaerobes

**DOI:** 10.1186/s12864-024-11128-x

**Published:** 2024-12-26

**Authors:** Rubén Javier-López, Natia Geliashvili, Nils-Kåre Birkeland

**Affiliations:** 1 Department of Biological Sciences, University of Bergen, Bergen, N-5020 Norway; 2 Present address: Institute of Biodiversity, Friedrich Schiller University Jena, Jena, 07745 Germany

**Keywords:** Comparative genomics, Fervidobacterium, Biotechnology, Horizontal gene transfer, Pan-genome, Phylogeny, Next generation sequencing, Genome assembly, Genome annotation

## Abstract

**Background:**

*Fervidobacterium* is a genus of thermophilic anaerobic Gram-negative rod-shaped bacteria belonging to the phylum *Thermotogota*. They can grow through fermentation on a wide range of sugars and protein-rich substrates. Some can also break down feather keratin, which has significant biotechnological potential. Fervidobacteria genomes have undergone several horizontal gene transfer events, sharing DNA with unrelated microbial taxa. Despite increasing biotechnological and evolutionary interest in this genus, only seven species have been described to date. Here, we present and describe six new and complete *Fervidobacterium* genomes, including the type strains *Fervidobacterium gondwanense* CBS-1^ T^, *F. islandicum* H-21^ T^ and *F. thailandense* FC2004^T^, one novel isolate from Georgia (strain GSH) and two strains (DSM 21710 and DSM 13770) that have not been previously described along with an evolutionary and phylogenomic analysis of the genus.

**Results:**

The complete genomes were around 2 Mb with approximately 2,000 CDS identified and annotated in each of them and a G + C content ranging from 38.9 mol% to 45.8 mol%. Phylogenomic comparisons of all currently available *Fervidobacterium* genomes, including OrthoANI and TYGS analyses, as well as a phylogenetic analysis based on the 16S rRNA gene, identified six species and nine subspecies clusters across the genus, with a consistent topology and a distant and separately branching species, *Fervidobacterium thailandense*. *F. thailandense* harbored the highest number of transposases, CRISPR clusters, pseudo genes and horizontally transferred regions The pan genome of the genus showed that 44% of the genes belong to the cloud pangenome, with most of the singletons found also in *F. thailandense.*

**Conclusions:**

The additional genome sequences described in this work and the comparison with all available *Fervidobacterium* genome sequences provided new insights into the evolutionary history of this genus and supported a phylogenetic reclassification. The phylogenomic results from OrthoANI and TYGS analyses revealed that *F. riparium* and *F. gondwanense* belong to the same genome species, and includes *Fervidobacterium* sp. 13770, while “*F. pennivorans*” strain DYC belongs to a separate genome species, whereas *Fervidobacterium* sp. 21710 and *Fervidobacterium* sp. GSH within the *Fervidobacterium pennivorans* clade represent two subspecies. *F. changbaicum* is reclassified as *F. islandicum*.

**Supplementary Information:**

The online version contains supplementary material available at 10.1186/s12864-024-11128-x.

## Background

*Fervidobacterium* is a genus of thermophilic and anaerobic bacteria belonging to the phylum *Thermotogota*, order *Thermotogales*, family *Fervidobacteriaceae*. All members of this phylum share a particular morphological characteristic; they are surrounded by an external sheath-like membrane called toga, which is a defining characteristic of the phylum [1, 2]. Previous studies have shown that some members of *Thermotogales* share up to 11% of their genes with diverse prokaryotic taxa such as Archaea, *Firmicutes*, and *Aquificae*, which inhabit the same high-temperature environments [3]. This confirms that the genomes of *Thermotogae* species have undergone extensive horizontal gene transfer events with other microbial groups [4–6], which has hindered the correct phylogenetic placement of these bacteria in the tree of life [7, 8].

Members of the phylum *Thermotogota* are currently classified into four orders (*Kosmotogales*, *Mesoaciditogales*, *Petrotogales*, and *Thermotogales*) and five families (*Kosmotogaceae, Mesoaciditogaceae, Petrotogaceae, Thermotogaceae*, and *Fervidobacteriaceae*). A total of 72 genomes, including isolates and metagenomes, are available. The family *Fervidobacteriaceae* is divided into two genera, *Thermosipho* and *Fervidobacterium* (https://www.ncbi.nlm.nih.gov/Taxonomy/Browser/wwwtax.cgi; accessed on December 5, 2023). A total of 29 *Fervidobacterium* genome sequences, including metagenome-assembled genomes (MAGs), were found in the GenBank database (https://www.ncbi.nlm.nih.gov/datasets/taxonomy/2422/; accessed November 27, 2023). The average genome size of Fervidobacteria is 2 Mb, with a G + C content ranging from 32 to 46% [9]. However, only seven *Fervidobacterium* species have been described to date: *Fervidobacterium nodosum* (CP000771.1) [10], *F. islandicum* [11], *F. gondwanense* (NZ_FRDJ00000000.1) [12], *F. pennivorans* (CP003260.1) [13], *F. changbaicum* CBS-1^T^ (CP026721.1) [14], *F. riparium* (CP009277.1) [15], and *F. thailandense* (NZ_LWAF00000000.1) [16].

Fervidobacteria are Gram-negative fermentative rod-shaped bacteria that thrive at temperatures ranging from 65 to 80 °C [10–16]. Like the rest of *Thermotogota*, they can grow on a variety of sugars and protein-rich substrates [7] and have been isolated from terrestrial hot springs worldwide [11–16]. Notably, some members of the genus *Fervidobacterium*, namely *F. pennivorans* DSM9078^T^ [13], *F. islandicum* AW-1 [17], *F. thailandense* FC2004C^T^ [16] and, more recently, *F. pennivorans* T [9] have been found to degrade feather keratin at high temperatures. As feather keratin is an underutilized agricultural byproduct and its current processing methods are inefficient, this metabolic ability has a significant biotechnological potential [18–21].The present study aims to describe six new *Fervidobacterium* genomes and presents an extensive evolutionary analysis of this underexplored genus. This reveals the presence of several genomic islands, horizontally transferred genes, and multiple transposase genes, as previously reported in this group [4]. Furthermore, genes for a number of different carbohydrate-active enzymes were identified in the studied genomes. The construction of a pan-genome, including all available genomes of the genus *Fervidobacterium*, along with whole genome-based and 16S rRNA gene-based phylogeny reconstructions, contributes with new insights into the evolution and phylogenetic relationships of this group of bacteria.

## Materials and methods

### Strains used in this study

The complete genomes of six *Fervidobacterium* species were sequenced and compared with previously known genomes, making a total of 17 genomes and MAGs being used in this study. Four strains were obtained from the German Collection of Microorganisms and Cell Cultures (Leibniz Institute DSMZ, https://www.dsmz.de), namely *F. gondwanense* DSM13020^T^, *F. islandicum* H-21^T^, and two uncharacterized strains: *Fervidobacterium* sp. DSM 21710 and *Fervidobacterium* sp. DSM 13770.*F. thailandense* FC2004^T^ was acquired from the Japan Collection of Microorganisms (JCM, https://jcm.brc.riken.jp).

*F*. *pennivorans* strain GSH was isolated from a water sample obtained from a hot borehole spring in Shakshaketi, Georgia (42.066570°E, 43.788732°N). The temperature and pH of the water were 75 °C and 6.5–7.0, respectively. Samples were collected in sterile serum flasks that were tightly sealed with butyl rubber stoppers and aluminum crimps and transported at ambient temperature to Bergen, Norway. Enrichment was performed in anaerobic serum flasks as described below, and the GSH strain was isolated using dilution-to-extinction.

The following genomes of the already described isolates were incorporated to the genome pull used in this study: *F. nodosum* Rt17-B1^T^ (CP000771.1), *F. islandicum* AW-1 (CP014334.2), *F. pennivorans* DSM9078^T^ (CP003260.1), *F. pennivorans* DYC (CP011393.1), *F. changbaicum *CBS-1^T^ (CP026721.1), *F. riparium* 1445t^T^ (CP009277.1), and *F. pennivorans* strain T (CP050868.1).

Additionally, the dataset was augmented with the following four MAGs and genomes of fervidobacteria downloaded from the National Center for Biology Information (NCBI) genome database (https://www.ncbi.nlm.nih.gov/datasets/genome) and used for comparative analyses: The MAGs *Fervidobacterium* sp. Ch94 (GCA_013177925.1) and *Fervidobacterium* sp. RSWP18 (GCA_028276185.1), the isolate genome *Fervidobacterium* sp. 2310opik-2 (GCF_011057735.1) and the single cell genome *Fervidobacterium* sp. SC_NGM5_G05 (GCA_002600545.1). These MAGs and genomes had more than 90% completeness and less than 5% contamination as assessed by CheckM [22], possessed at least 18 tRNA genes, and were confirmed to represent *Fervidobacterium* by the Microbial Genomes Atlas (MiGA) online server [23, 24]. Thus, these MAGs and genomes were considered to be of high quality and were included in the study [25].

### Cultivation

A common medium named MMF (mineral medium for freshwater bacteria) was used for cultivation. This medium had the following composition, per liter: NaCl, 1 g; MgSO_4_·7H_2_O, 0.3 g; KCl, 0.3 g; NH_4_Cl, 0.5 g; CaCl_2_·2H_2_O, 0.1 g; and KH_2_PO_4_, 0.3 g. Additionally, 1 mL of SL-10 trace minerals [26] and 0.5 g of yeast extract were added. This mixture was autoclaved at 121 °C for 20 min and cooled to 60 °C while being gassed with sterile nitrogen. Then, 10 mL of a vitamin solution was added, containing the following components: 4-aminobenzoic acid, 8 mg/L; D( +) biotin, 2 mg/L; nicotinic acid, 20 mg/L; Ca-D( +) pantothenic acid, 10 mg/L; pyridoxamine·2HCl, 30 mg/L; thiamine dichloride, 20 mg/L; and vitamin B12, 10 mg/L. A reducing agent (cysteine HCl) was added at a final concentration of 0.5 g per liter. The pH of the final medium was adjusted to 7 using 1 M HCl. Finally, 100 mL serum flasks were aseptically filled with this medium following the Hungate technique [27, 28], closed with butyl rubber stoppers, and sealed with aluminum crimp caps. Peptone (final concentration 5 g/L) was used as the primary carbon source. Each strain was incubated at their optimal temperature according to DSMZ or JCM.

### Genomic DNA isolation

After overnight incubation in the MMF medium with peptone, the bacteria were harvested through centrifugation at 5000 rpm for 10 min at 4 °C. Genomic DNA was purified using the GenElute™ Bacterial Genomic DNA Kit (Sigma-Aldrich, St. Louis, MO, USA) following the standard protocol recommended by the manufacturer for Gram-negative bacteria with minor modifications. The lysis incubation step was extended to 60 min at 65 °C to ensure optimal DNA extraction. The DNA concentration and purity were assessed using a NanoDrop™ One/One^C^ spectrophotometer. Furthermore, 1% agarose gel electrophoresis was performed to evaluate the quality and integrity of genomic DNA.

### Genome sequencing and assembly

Genomic DNA was sequenced at Eurofins Genomics, Constance, Germany (https://eurofinsgenomics.eu/), using Oxford Nanopore (ONT) and Illumina technologies. Raw Illumina HiSeq 2000 reads for *F. gondwanense* DSM13020^T^ (SRR4096409) were downloaded from SRA Explorer (https://sra-explorer.info) to polish the long reads. *F. thailandense* FC2004^T^ was sequenced in-house. A NEBNext® Ultra™ II DNA Library Prep Kit for Illumina® (New England Biolabs, Ipswich, MA) was used to prepare the libraries, and sequencing was carried out using a MiniSeq™ sequencing system (Illumina, San Diego, CA). Nanopore raw reads were trimmed by Filtlong [29] using Illumina reads as a reference. The 10% lowest-quality reads and those shorter than 1000 bp were discarded. Similarly, the Illumina reads were trimmed using BBDuk [30], removing adapters and low-quality reads (PHRED scores < 20). The trimmed reads from both the ONT and Illumina sequencing projects were hybrid-assembled and polished using Unicycler v0.5.0 [31], excluding contigs shorter than 200 base pairs, with the rest of the options set by default.

### Gene prediction and genome annotations

All six genomes were annotated using the Prokaryotic Genome Annotation Pipeline (PGAP) version 2023-05-17.build6771 [32] (https://github.com/ncbi/pgap) and Prokka v1.4.15 [33]. The Prokka default libraries were enhanced by adding the following extra hidden Markov model databases: PFAM [34], PGAP v12.0, and TIGFRAMs [35].

### Phylogenetic and phylogenomic analyses

The 16S rRNA gene sequences of the *Fervidobacterium* cohort were aligned and used to construct a Maximum Likelihood phylogenetic tree using the Mega11 software suite [36].

All the genomes of these bacteria plus additional *Fervidobacterium* genomes and MAGs with completion estimates ≥ 90% and ≤ 5% contamination were included in the phylogenomic analyses. The overall similarity between each pair of genomes was determined using the Orthologous Average Nucleotide Identity (OrthoANI) algorithm using OAU (OrthoANI tool using the USEARCH algorithm) [37]. Additionally, genome-based phylogeny was inferred and dDDH (digital DNA-DNA Hybridization) estimated using the Type (Strain) Genome Server (TYGS) [38]. The pairwise dDDH values were calculated using the GGDC formula 2 (d_4_), sum of all identities found in HSPs (High-scoring Segment Pairs) divided by overall HSP length [39].

### Genomic comparisons

The annotated assemblies from the Prokka software tool were used to construct a pan-genome using the Roary pipeline version 3.13.0 [40]. All *Fervidobacterium* genomes and MAGs mentioned above, i.e., both the experimental strains and those downloaded from NCBI, were included in this analysis, resulting in a database comprising 17 different genomes. For a gene to be considered a part of the core pan-genome, it had to be present in at least 99% of the genomes, with a BLASTP identity threshold of at least 80%. Additionally, another pan-genome was constructed using Anvi’o pipeline with a Markov Cluster Inflation (MCL) parameter value of 6 [41, 42].

The studied genomes were uploaded to the Phage search tool-enhanced release (Phaster) [43, 44] web server for annotation and identification of putative prophage sequences.

Additionally, the studied *Fervidobacterium* genomes were scanned using IslandViewer [45] and Alien Hunter (sanger.ac.uk) to detect genomic islands and regions of horizontal gene transfer. The SIGI-HMM, Islandpick, Islander, and Island Path-DIMOB methods were used to find genomic islands within the studied genomes. All annotations were assembled and analyzed using Proksee [46].

CRISPRCasFinder [47–49] was used to detect CRISPR/Cas9 clusters. The general method provided by the service was chosen, while keeping the remaining parameters default.

### Functional annotations

Orthologous gene cluster relationships and orthogroup detection in the genus *Fervidobacterium* were conducted using the Orthofinder software [50, 51]. Gene functions in the *Fervidobacterium* strains were annotated and classified using the cluster of orthologous groups (COGs) [52] by scanning the genomes with the COG classifier tool [53]. The global metabolism of the different strains was analyzed using the Kyoto Encyclopedia of Genes and Genomes (KEGG) database [54–56] and explored using iPATH3 [57]. Finally, the Automated Carbohydrate-active enzyme and substrate ANnotation (dbCAN3) web server (https://bcb.unl.edu/dbCAN2/) was used at default values to identify and annotate the carbohydrate-active enzymes (CAZy) across the genomes [58] whose proteomes were annotated using HMMER via dbCAN.

## Results

### Genome assemblies and annotations

The six strains sequenced individually using GridION Oxford Nanopore technology yielded 3.4 Gb of data in total, with genome coverage ranging from 195 × to 319x. After trimming with Filtlong, the coverage decreased, ranging from 176 × to 252x (Table S1). Likewise, the Illumina sequencing projects yielded 9.48 Gb of data in total, with genome coverage from 199 × to 1,094 × before trimming and from 192 × to 1,022 × after trimming with BBDuk (Table S1).

The trimming and hybrid assembly yielded complete sequences corresponding to bacterial chromosomes, each with a single contig of approximately 2 Mb. No plasmids were detected. After genome annotation with the Prokaryotic Genome Annotation Pipeline (PGAP) the six genomes were deposited in GenBank where the following accession numbers were assigned: *F*. *pennivorans* GSH (CP126982), *Fervidobacterium* sp. 13770 (CP126498), *F. islandicum* H-21^T^ (CP126499), *Fervidobacterium* sp. 21710 (CP126500), *F. gondwanense* DSM13020^T^ (CP126501), and *F. thailandense* FC2004^T^ (CP140110).

All genomes were of similar size (approximately 2 Mb) (Table 1). PGAP identified approximately 2,000 coding regions in all genomes, 57–58 of which were annotated as RNA genes. The G + C content was approximately 40 mol% in all strains except for *F. thailandense* FC2004^T^, which had a G + C content of 45.8 mol%. The number of CRISPR/Cas arrays varied from two for strains 21710 and GSH to ten for *F. thailandense* FC2004^T^ (Table 1). The genomes encode different numbers of transposases, ranging from three in *F. gondwanense* DSM13020^T^ to 32 in *F. thailandense* FC2004^T^.

**Table 1:** General characteristics of the genomes of the *Fervidobacterium* strains described in this study^a^

	*F.* sp. 13770	*F.* sp. 21710	*F.* sp. GSH	*F. gondwanense* DSM13020^T^	*F. islandicum* H-21^T^	*F thailandense* FC2004^T^
Country of origin	Germany	Tunisia	Georgia	Australia	Iceland	Thailand
Year of isolation	Before 1987	2007	2019	Before 1997	Before 1990	2012
Genome size (bp)	2,140,048	2,102,275	2,023,003	2,176,180	2,192,603	2,074,176
N^o^ CDS with protein	1,978	1,973	1,871	1,984	1,987	1,891
Hypothetical proteins	256	251	261	254	266	244
Total RNAs	57	58	57	57	57	57
N^o^ rRNAs						
5S	2	3	2	2	2	2
16S	2	2	2	2	2	2
23S	2	2	2	2	2	2
tRNAs	48	48	48	48	48	48
ncRNAs	3	3	3	3	3	3
Pseudo genes	16	13	14	16	17	19
CRISPR clusters	4	5	2	6	6	10
Transposases	7	4	8	3	19	32
G + C content (mol%)	39.8	38.9	39.0	39.7	40.8	45.8
Genbank accession numbers	CP126498	CP126500	CP126982	CP126501	CP126499	CP140110

^a^Years of isolation were retrieved from the German Collection of Microorganisms and Cell Cultures (DSMZ). The genomic data were locally annotated using the PGAP pipeline. CRISPR clusters were identified using CRISPR Finder. Transposases were found using IslandViewer

**Fig. 1:**
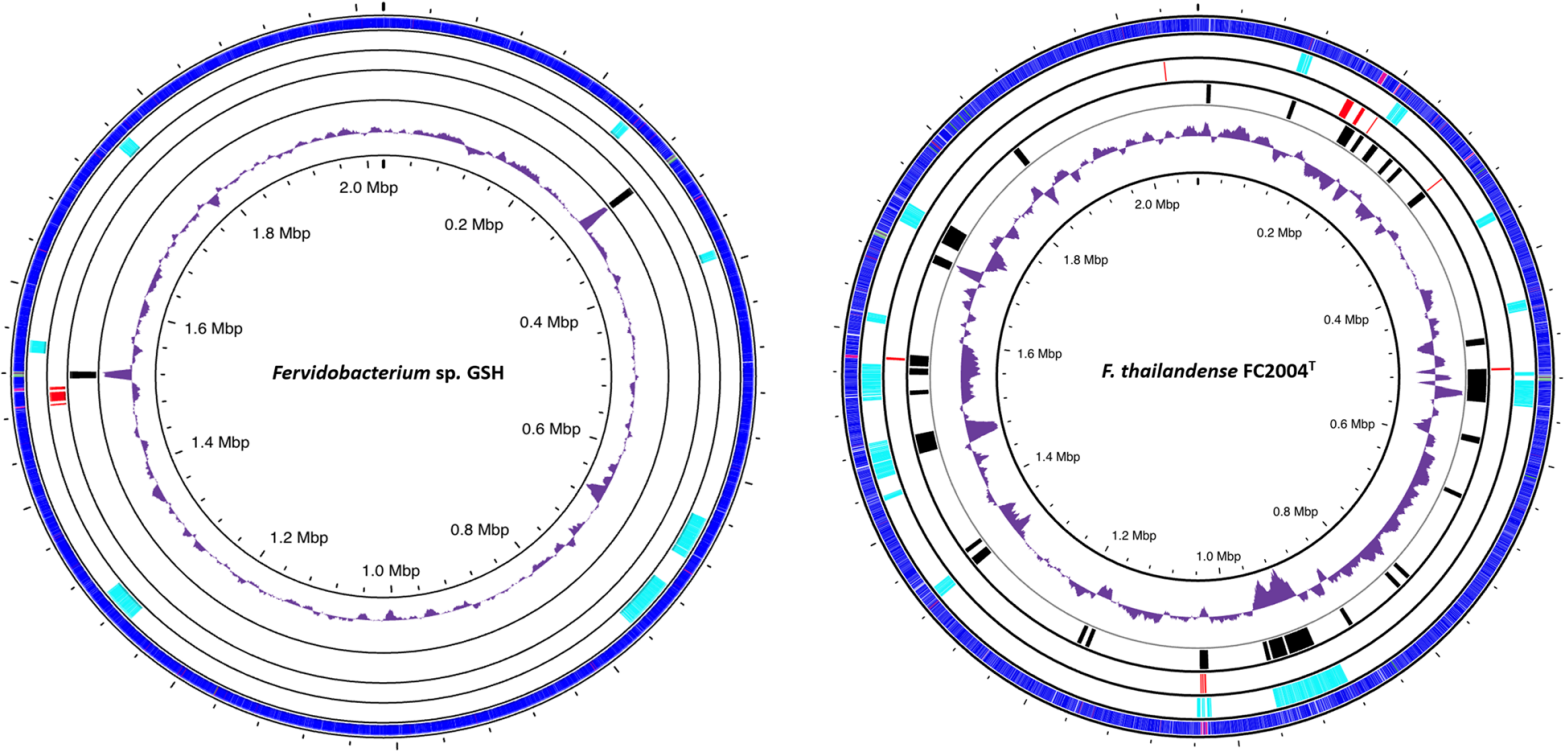
Different features annotated in fervidobacteria described in this study. Each set of rings represents the analyzed genomes. From inner to outermost: backbone (black), G + C content (purple), regions of horizontal gene transfer predicted by Alien Hunter (black), CRISPR/Cas9 clusters found by CRISPR/CasFinder (red), genomic islands annotated with IslandViewer (skyblue), and coding sequences (blue). The figure was made using Proksee

IslandViewer predicted a variable number of regions that probably are horizontally transferred in the form of genomic islands. *F. thailandense* FC2004^T^ and *F. islandicum* H-21^T^ had the highest number of transposases and genomic islands predicted by this tool, with 13 and 12 fragments, respectively. The remaining strains had a lower number of genomic islands, ranging from four to seven. The genomes were also mapped using the Alien Hunter v1.7 [59] tool to predict putative horizontal gene transfer events. This analysis detected 30 features across the genome of *F. thailandense* FC2004^T^, the largest number among the studied genomes. The number of features and regions found in the rest of the genomes was much lower; four fragments were detected in *F.* sp. 13770, *F.* sp. 21710, and *F. gondwanense* DSM13020^T^, and only two in *Fervidobacterium* sp. GSH, and *F. islandicum* H-21^T^ (Fig. 1). The positions of all these features in the genomes of *Fervidobacterium* sp. GSH and *F. thailandense* FC2004^T^ are shown in Fig. 1, and those of the other genomes in Figure S1. The genomic islands and their annotations found by IslandViewer are presented in a separate Excel file (Supplementary Table S2).

The presented genomes were all similar in size, number of CDS and RNAs. However, *F. thailandense* FC2004^T^ showed the highest number of mobility-related features: transposase genes and CRISPR clusters, which can at least partially explain the divergent G + C content in its genome.

### Pan-genome

The pan-genome scale comparison conducted using Roary included the six genomes sequenced in this study and additional complete and available genomes downloaded from the GTDB and NCBI. A total of 7,671 gene clusters were considered, 368 of which constituted the core pan-genome, indicating that these genes were present in the 17 organisms analyzed. The shell pan-genome included 3,929 genes found between 3 and 16 genomes. The cloud pan-genome comprised 3,374 genes present in two or only one bacterium, indicating that more than 44% of the genes in the fervidobacteria pan-genome were cloud genes (Figure S2).

**Fig. 2:**
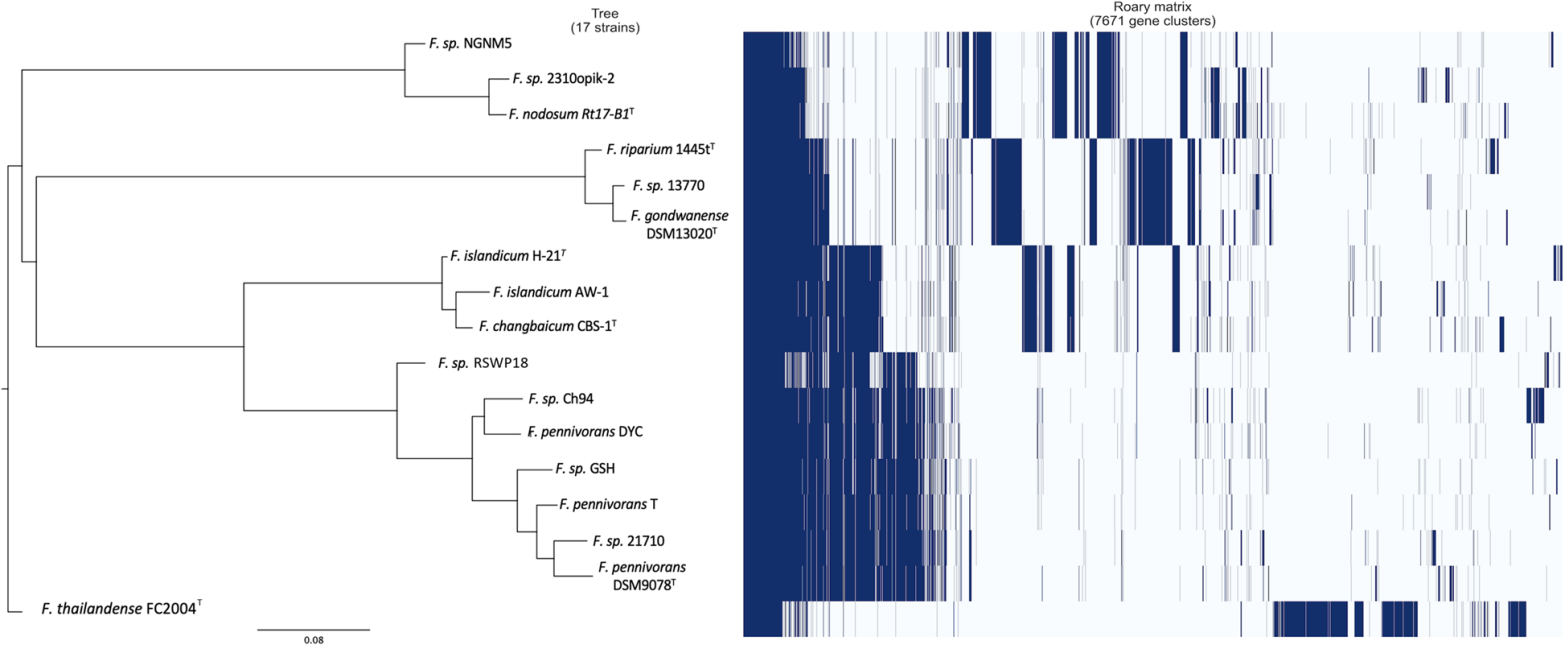
Pan-genome matrix and tree showing the 7,671 gene clusters analyzed and the 17 strains included in the analysis. Navy-blue color represents the gene clusters present in the corresponding strain, whereas the pale blue color represents the absent gene clusters. The figure was made using Roary plots script v.0.1.0 [60]

The gene presence/absence matrix and the phylogenetic tree constructed using Roary were combined (Fig. 2). The topology of the ortholog cluster-based tree was similar to that of the genome-based tree (Fig. 4), with six clusters already described. *Fervidobacterium* sp. strain NGM5 and the single-cell genome *F.* sp. 2310opik were associated with *F. nodosum *Rt17-B1^T^, indicating that *F.*sp. 2310opik had an almost identical ortholog profile to that of *F. nodosum *Rt17-B1^T^.* F. riparium*1445t^T^ shared another cluster with *F.*sp. 13770 and *F. gondwandense* DSM13020^T^, which were found to be even closer to each other with almost identical genetic profiles in the Roary matrix. *F. islandicum* H-21^T^ and AW-1 formed another cluster along with *F. changbaicum* CBS-1^T^, and their genetic profiles in the matrix were almost identical. *F. pennivorans* DYC and *F.* sp. Ch94 were placed together in the tree, and *F.* sp. RSWP18 was placed next to them. These three strains were somewhat integrated in the *F. pennivorans* cluster but were closer to each other than to the species type strain *F. pennivorans* DSM9078^T^. The remaining *F. pennivorans* strains, namely *F*. sp. GSH, *F. penni*vorans T, and *F*. sp. 21710, clustered together with the species type strain, remaining more distant from *F. pennivorans* DYC, *F*. sp. Ch94 and *F.* sp. RSWP18. Finally, *F. thailandense* FC2004^T^ branched off, forming an independent cluster with an ortholog profile different from that of the other members of the group.

The cloud genome counted 248 transposase related features, 33 of which were exclusively found in *F. thailandense* FC2004^T^. Furthermore, 13 CRISPR-related proteins were unique of *F. thailandense* FC2004^T^. Also, from a total of 2,709 singletons found in the pan genome, 1,352 belonged to this strain. This may explain the genomic differences between this organism and the rest of the members in the genus.

The pan genome constructed with Anvi’o (Figure S3) gave similar results regarding the core genome, with 366 singleton gene clusters found in 1,999 gene clusters in a total of 33,188 genes. Also, most of the singletons were detected in *F. thailandense* FC2004^T^.

### Phylogeny

**Fig. 3:**
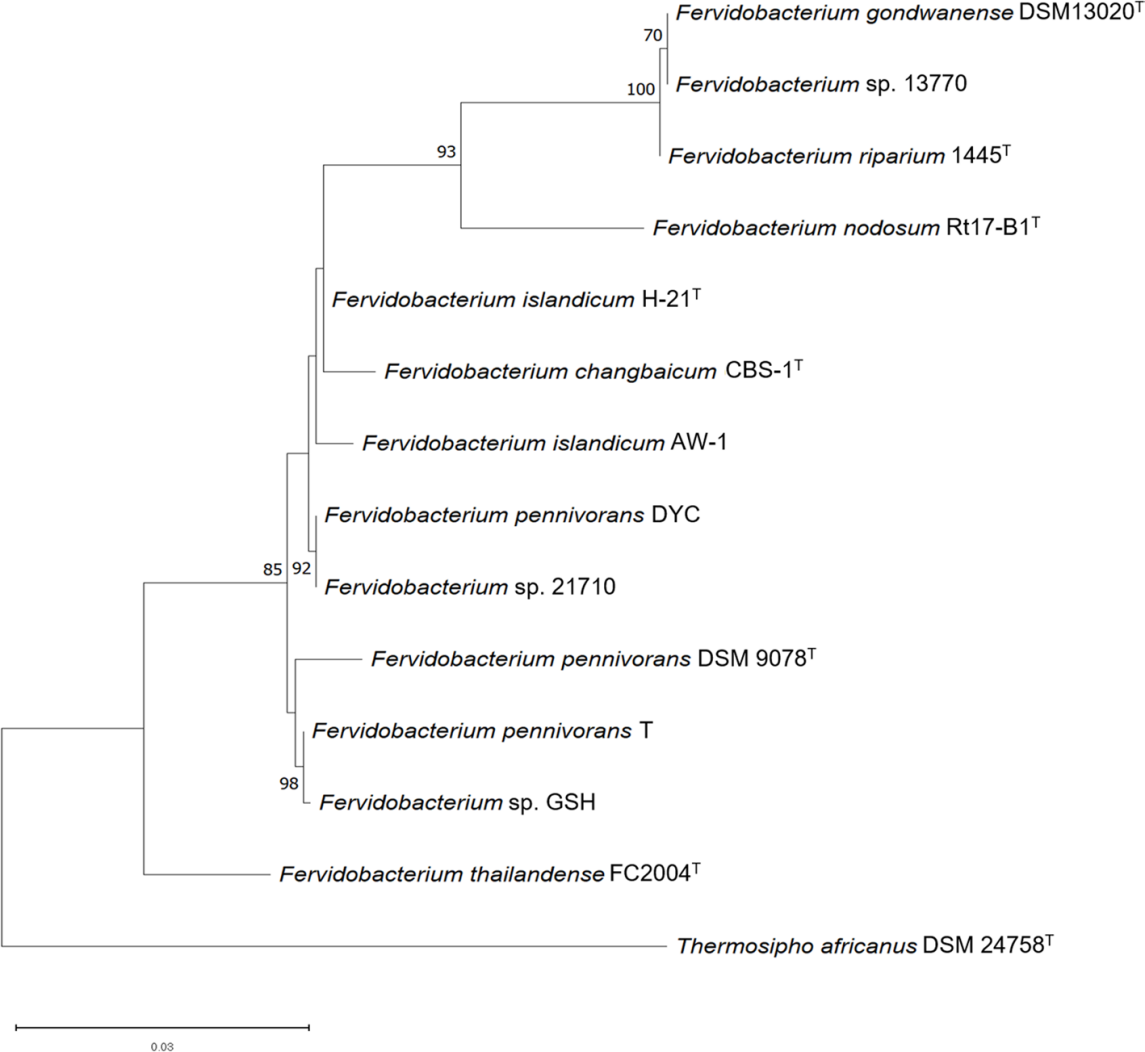
Maximum likelihood phylogenetic tree showing the evolutionary history of the genus Fervidobacterium based on the 16S rRNA gene. *Thermosipho africanus* DSM 24758 was used as an outgroup. The tree with the highest likelihood (-3,183.75) is shown, with bootstrap values after 1000 replicates. This analysis included 14 nucleotide sequences. All positions containing gaps and missing data were eliminated (complete deletion option). The final dataset contained a total of 1,304 positions

The maximum likelihood-based phylogenetic tree inferred for the 14 *Fervidobacterium* organisms whose 16S rRNA gene sequences were available is shown in Fig. 3. Strain 13770 was placed together with *F. gondwanense* DSM13020^T^ and *F. riparium* 1445t^T^ with high bootstrap value. The 16S rRNA gene identities fell within the species thresholds: 99.67% to 99.80%. *F. islandicum* H-21^T^ and *F. islandicum* AW-1 strains shared 98.27% identity, and compared with *F. changbaicum* CBS-1^T^, they shared 98.27% and 99.07% identity, respectively, indicating that these three strains constitute a distinct species cluster. *F.* sp. 21710 was affiliated with *F. pennivorans* DYC, sharing an almost identical 16S rRNA gene sequence (99.47%). Strain GSH was affiliated with *F. pennivorans* T, sharing 99.73% identity, and conformed a species cluster with the type strain, *F. pennivorans* DSM 9078^T^. Compared with the species type strain, the 16S rRNA gene identity was 98.53% for *F. pennivorans* GSH and 98.19% for *F.* sp. 21710. Finally, *F. thailandense* FC2004^T^ was not associated with any of the described clusters, constituting an independent branch, with identity values ranging from 94.36% with *F. nodosum* Rt17-B1^T^ GSH to 96.62% with *F. pennivorans* T.

**Fig. 4:**
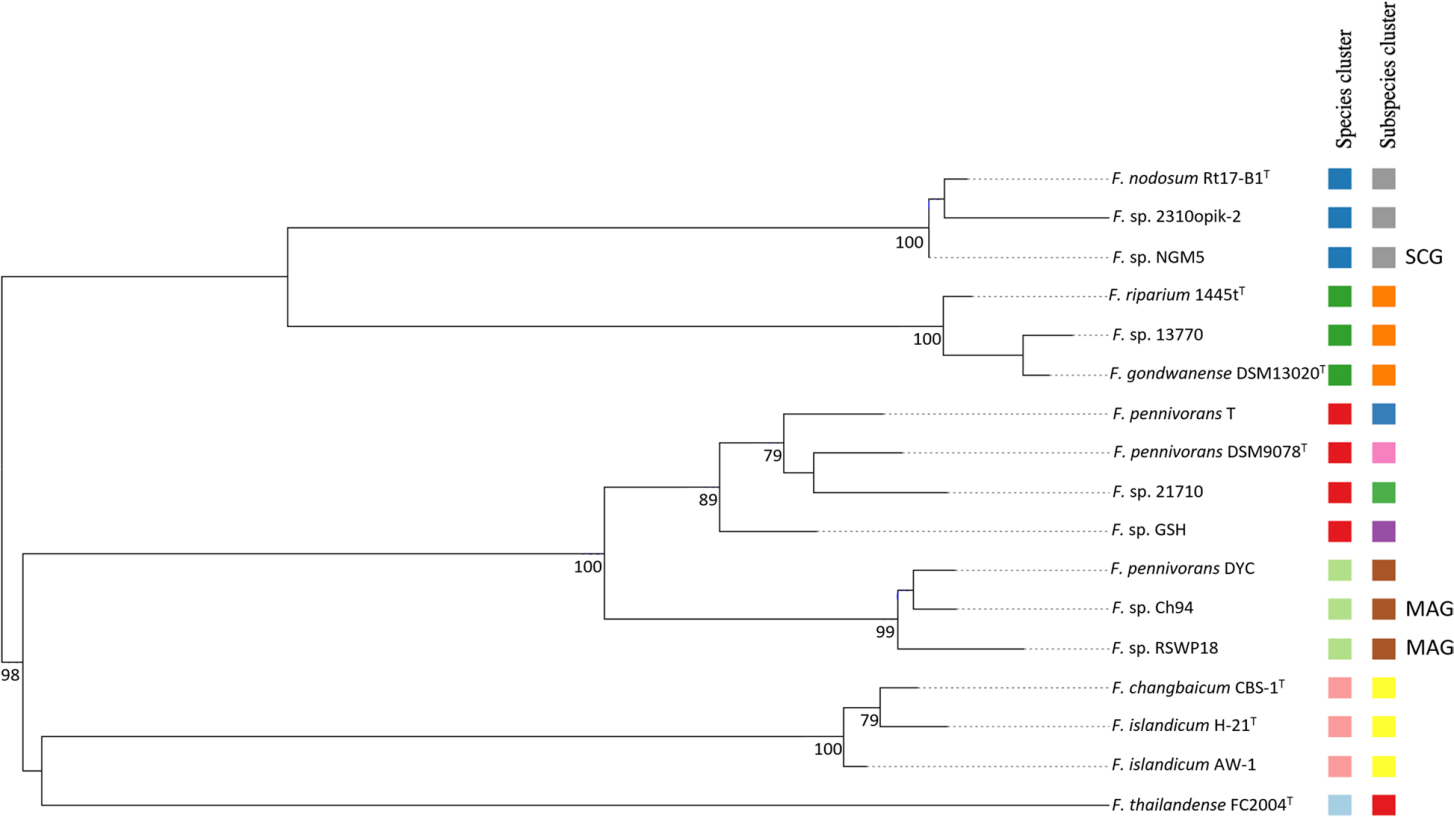
Phylogenomic tree of the* Fervidobacterium* species, strains, metagenome-assembled genomes (MAGs), and single-cell genome (SCG) constructed using the TYGS genome server (https://tygs.dsmz.de). The tree was inferred with FastME 2.1.6.1 [62] from Genome Blast Distance Phylogeny (GBDP) distances calculated from the genome sequences, with an average branch support of 81.1%. The branch lengths are scaled in terms of GBDP distance formula d5. The numbers at branches are GBDP pseudo-bootstrap support values ≥ 60% from 100 replications. The tree was rooted at midpoint [63]. The genome sequence accession numbers are as follows: * F. gondwanense*, CP126501; *F. nodosum*, GCA_000017545; *F*. sp. 2310opik-2, GCA_011057735.1; *F*. sp. NGM5, GCA_002600545; * F. pennivorans* strain DYC, GCA_001644665.1; * F. pennivorans* DSM 9078, GCA_000235405; *F. pennivorans* subsp. *keratinolyticus* strain T, CP050868; *F. changbaicum*, GCA_900100515; *F. islandicum* AW-1, GCA_000767275.4; *F thailandense*, CP140110

This grouping was confirmed by the genome-based phylogenetic tree reconstructed using the TYGS server (Fig. 4). For this comparison, the aforementioned MAGs and genomes downloaded from NCBI were also included. This tree showed a branching pattern similar to that of the 16S rRNA-based tree, with six well-differentiated species clusters and nine subspecies clusters. *F. nodosum* Rt17-B1^T^, *Fervidobacterium* sp. 2310opik, and *Fervidobacterium* sp. NGM5 comprised one species cluster, with dDDH values of 79.3% and 96.8%, respectively, comparing *F. nodosum* Rt17-B1^T^ with these genomes, according to the GGDC formula 2 (d_4_), with an estimated identity between the two latter genomes (*Fervidobacterium* sp. 2310opik and *Fervidobacterium* sp. NGM5) of 80%. *Fervidobacterium* sp. 13770 clustered with *F. gondwanense* DSM13020^T^ and *F. riparium* 1445t^T^, with dDDH values as high as 92.1% and 86.6%, respectively. The species grouped as *F. pennivorans* were *F. pennivorans* DSM9078^T^, *Fervidobacterium* sp. 21710, with a dDDH of 75.8%, and *F. pennivorans* T and *F*. sp. GSH, which shared 76.7% and 70.2%, respectively, with *F. pennivorans* DSM9078^T^, and 71.4% and 66.2%, respectively, with *F*. sp. 21710. It should be noted that each strain in this *F. pennivorans* group scored as separate subspecies, since all pairwise dDDH values were lower than 79%, subspecies boundary according to TYGS [61]. Strain DYC, previously classified as *F. pennivorans*, grouped together as a separate species cluster with *Fervidobacterium* sp. RSWP18 and *F.* sp. Ch94. *F. islandicum* H-21^T^, *F. islandicum* AW-1, and *F. changbaicum* CBS-1^T^ conformed another independent genome species cluster with a minimum pairwise dDDH value of 87.4%, indicating that they belong to the same genome species. Finally, *F. thailandense* FC2004^T^ branched independently from the rest, as in the 16S rRNA-based tree, with a dDDH value as low as 17.2% when compared to *Fervidobacterium* sp. Ch94, and 21.8% dDDH compared to *Fervidobacterium* sp. 21710.

**Fig. 5:**
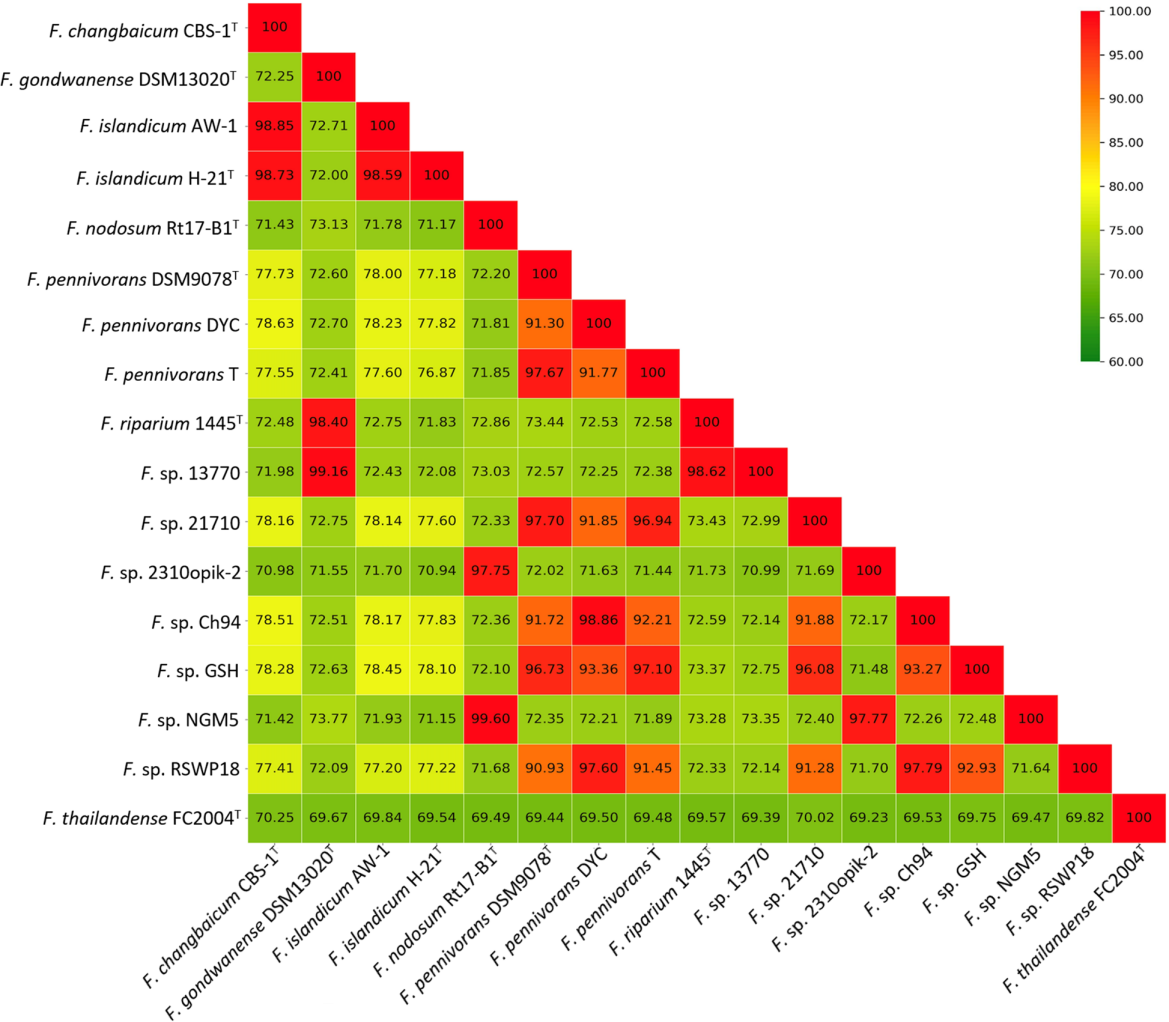
OrthoANI matrix with values calculated using the genome sequences of the members of the genus *Fervidobacterium*. The values represent the overall similarities between each pair of genomes. The species cut-off was set at 95%

OrthoANI values were also calculated for all available genomes of the group. Genomes with values over 95% belong to the same species [64]. The previously described relationships were confirmed by this analysis. *F. islandicum* H-21^T^ was placed closer to *F. changbaicum* CBS-1^T^ (OrthoANI value of 98.73%) compared to *F. islandicum* AW-1 (98.59%), and all three in the same species group. Likewise, *F*. sp. 13770 was again associated with *F. gondwanense* DSM13020^T^ (99.16%) and *F. riparium* 1445t^T^ (98.62%). The OrthoANI value for the latter two was 98.40%. The strains closest to *F*. sp. 21710 were *F. pennivorans* DSM9078^T^ (97.7%), *F. pennivorans* T (96.94%) and *F*. sp. GSH (96.08%), all of them above the species threshold value. Again, *F. thailandense* FC2004^T^ was the most distant strain compared to the rest of the *Fervidobacterium* members, with OrthoANI values lower than 70.25%. A heat map with the OrthoANI values is shown in Fig. 5.

Overall, all the phylogenetic and phylogenomic analyses showed equivalent but complementary results. Furthermore, the topology of the TYGS and OrthoANI trees is congruent and reinforces the proposed topology and phylogenetic clustering of the genus, implicating the necessity for taxonomic reclassification of *F. riparium* and *F. changbaicum*.

### Functional annotations and metabolic predictions

The COG classifier annotated 83–85% of the protein-coding genes from each of the studied fervidobacteria using the COG database. Among the COG categories, a large number of genes were related to metabolism, with clusters corresponding to carbohydrate and amino acid transport and metabolism being the predominant features in all strains, something expected in fermentative organisms. The most abundant COG functions for all genomes were translation, ribosomal structure, and biogenesis, with more than 180 orthologous groups found in all the genomes described in this study (Figure S4). Again, the genome characteristics related to gene mobility (mobilome) were more abundant in *F. thailandense* FC2004^T^ compared to the other organisms. Approximately 40 clusters (2.55–2.95% of the sequences) with unknown functions remained for each genome after annotation.

Orthogroups were identified and annotated using the OrthoFinder algorithm. OrthoFinder scanned 17 species, analyzed 31,582 genes, and found 2,258 orthogroups, 1,021 had all the species present. Only 1.1% of the genes remained unassigned (Table 2). In the strains described in this study, almost 100% of the genes were assigned to orthogroups. *F. thailandense* FC2004^T^ had the highest percentage of unassigned genes (2.8%) and the highest percentage of species-specific orthogroups (1.4%), highlighting once more the differences of this bacterium compared with the rest.

**Table 2:** Orthofinder output of the analyzed strains

	*F.* sp. 13770	*F.* sp. 21710	*F.* sp. GSH	*F. gondwanense* DSM13020^T^	*F. islandicum* H-21^T^	*F. thailandense* FC2004^T^	
Genes	1,978	1,973	1,871	1,984	1,987		1,891
Genes in orthogroups	1,968	1,957	1,863	1,969	1,970		1,838
Unassigned genes	10	16	8	15	17		53
Genes in orthogroups (%)	99.5	99.2	99.6	99.2	99.1		97.2
Percentage of unassigned genes	0.5	0.8	0.4	0.8	0.9		2.8
Orthogroups containing species	1,901	1,851	1,780	1,899	1,873		1,703
Orthogroups containing species (%)	84.2	81.9	78.8	84.1	82.9		75.4
Number of species-specific orthogroups	0	0	0	0	3		7
Genes in species-specific orthogroups	0	0	0	0	6		26
Genes in species-specific orthogroups (%)	0	0	0	0	0.3		1.4

All six newly sequenced fervidobacteria possessed similar numbers of CAZy genes (Table 3). The total number of these enzymes ranged from 58 in *F. thailandense* FC2004^T^ to 68 in *F.* sp. 21710. The most abundant CAZy categories were glycoside hydrolases (GH) and glycoside transferases (GT). The identified GH and their sequences can be found attached in a separate file (supplementary file S2). Only four or five carbohydrate esterases were found in each genome. *F.* sp. 21710 was the only strain with CAZy annotated as auxiliary activity (AA). Polysaccharide lyases were not detected in any of the genomes. The number of carbohydrate-binding modules (CBMs) varied from four to six for all fervidobacteria except *F. thailandense* FC2004^T^, which had only one CBM. *F. pennivorans* T was also included in the comparison. This strain showed a total of 68 CAZy and a categorization similar to that of the other members of the *F. pennivorans* cluster (*F.* sp. GSH and *F.* sp. 21710).

**Table 3:** Major carbohydrate- active enzymes (CAZy) categories detected in the studied *Fervidobacterium* species, found using HMMER via dbCAN^a^

Species	Total CAZy	GT	GH	PL	CE	AA	CBMs
*F.* sp. 13770	65	14	42	0	5	0	4
*F.* sp. 21710	68	21	36	0	4	1	6
*F. islandicum* H-21^T^	63	15	38	0	5	0	5
*F.* sp. GSH	59	21	29	0	4	0	5
*F. gondwanense* DSM13020^T^	65	14	42	0	5	0	4
*F. thailandense* FC2004^T^	58	27	25	0	5	0	1
*F. pennivorans* T	68	23	32	0	4	0	3

^a^*GT* Glycoside transferases, *GH* Glycoside hydrolases, *PL* Polysaccharide lyases, *CE* Carbohydrate esterases, *AA* Auxiliary activity, *CBMs* Carbohydrate-binding modules

BlastKOALA (KEGG Orthology And Links Annotation) annotation of the studied strains was used to analyze their metabolism. Approximately 60% of the genes of each strain were assigned to KEGG Orthology categories after the BlastKOALA annotation. The glycolysis pathway was found in the annotations of all analyzed organisms, indicating that all of them were capable of obtaining energy from glucose and transforming it into pyruvate, which can later be used to form acetyl-CoA and acetate or lactate. The gluconeogenesis pathway was also complete in all of them, according to the KEGG annotation. However, the TCA cycle is disrupted in all the studied bacteria, with a characteristic ‘horseshoe’ shape. Complete annotations of the pentose phosphate pathway were also observed, meaning that all the organisms can obtain ribulose 6-P and NADPH. Comparing the studied bacteria in more detail, only *F.* sp. GSH and *F. thailandense* FC2004^T^ lack L-lactate dehydrogenase (K00016, 1.1.1.27), which reduces pyruvate to lactate, suggesting metabolic differences among these organisms in terms of pyruvate utilization. The different KEGG annotations were very similar overall, with only minor differences among the organisms. For instance, the carotenoid biosynthesis pathway, which is related to the metabolism of terpenoids and polyketides, was partially found only in *F. gondwanense* DSM13020^T^, whereas *F.* sp. 13770 lacks tryptophan synthase (KEGG KO6001/EC 4.2.1.20), part of the serine and tryptophan metabolism, and *F. thailandense* FC2004^T^ has some minor differences in galactose metabolism. Three cytoplasmic [FeFe] hydrogenases (one subunit form) (EC 1.12.7.2) and one cluster of a cytoplasmic three-subunit bifurcating hydrogenase (EC1.12.1.4) were identified in the genomes. Both types of hydrogenases are involved in hydrogen evolution as electron sinks during fermentation. Two mechanisms of nitrogen assimilation from ammonia were identified: glutamate dehydrogenase (EC 1.4.1.13) and coupled glutamine synthetase (EC 6.3.1.2)/ glutamate synthase (EC 1.4.1.13).

### Keratinolytic potential

As previously noted, some members of the *Fervidobacterium* genus have been reported to degrade keratin, one of the most abundant and resistant proteins on Earth. A combination of different enzymes, including proteases and reductases, has been proposed to contribute to keratin degradation [65–67]. Thus, the presence of these enzymes within microbial genomes can be a good indicator of keratinolytic potential. Our inspection of the genomes annotated with PGAP showed that all studied strains possessed between 50 and 53 candidate proteases and between 53 and 62 reductases that may play important roles in keratin degradation.

## Discussion

The genus *Fervidobacterium* currently comprises seven validly published species, despite the availability of almost 30 genome assemblies. All members of this genus have been isolated from terrestrial hot springs and are either thermophilic or hyperthermophilic bacteria [68]. Despite their similar metabolic characteristics and physicochemical growth conditions, no standardized growth medium was available for all fervidobacteria with four different recipes used in previous studies [26]. The formulation described here, utilizing glucose as the main carbon source, allowed the growth of all the investigated isolates and can probably be thus used as a common recipe for the genus.

All six bacteria were genome-sequenced using Oxford Nanopore and Illumina technologies, with hybrid-assembly, a strategy that yielded high-quality and complete genome sequences.

KEGG annotations of the sequenced genomes showed that these bacteria can utilize and synthesize glucose since both glycolysis and the gluconeogenesis pathways were complete, in agreement with the previously described central metabolism of the Thermotogota [2]. Furthermore, the high number of genes assigned by COG classifier to carbohydrate and amino acid catabolism highlights the substrate preferences of these bacteria. Additionally, more than 50 peptidases and oxidoreductases were found in the described genomes. Since some of the members of the genus can effectively break down feather keratin these enzymes can be further explored and may stand out as candidates for feather keratin degradation. Finally, four hydrogen-evolving hydrogenases were identified in the studied genomes, indicating a potential for biohydrogen production, a feature already reported for other Thermotogota species [69].

Our phylogenomic analyses revealed that *F*. sp. 13770 should be considered to belong to the *F. gondwanense* species cluster, with OrthoANI and dDDH values of 99.16% and 92.1%, respectively, but with minor metabolic and genomic differences according to the thresholds proposed by Chun et al. [70]. Similarly, *F.* sp. 21710 and *F.* sp. GSH were classified by the TYGS analysis as different subspecies of *F. pennivorans*, with a dDDH of 75.8% and 75.2%, respectively, compared to the species type strain *F. pennivorans* DSM9078^T^. The OrthoANI values were over 96%, supporting those from TYGS, grouping these three strains into the same species cluster. Notably, the isolate previously termed *F. pennivorans* DYC, shared only a 44.3% dDDH value with the type strain and an OrthoANI percentage of 91.3%. This divergence and clustering of strain DYC with the two MAGs (Ch94 and RSWP18) suggested a separate species cluster and reclassification of *F. pennivorans* DYC as a novel species. Similarly, *F. changbaicum* shares substantial genomic similarity with *F. islandicum* strains H-21^T^ and AW-1 and forms a tight phylogenomic cluster. *F. changbaicum* should therefore be re-classified as *F. islandicum*. Furthermore, while *F. riparium* shows some phenotypical and physiological particularities and conformed an independent species cluster [15] it should be reclassified as a *F. gondwanense* strain because they form a tight species cluster which also includes strain DSM13770. Our results also show that *F. thailandense* FC2004^T^ is a distant species compared to the other members of the genus. This organism got the lowest values both in dDDH and OrthoANI calculations, raising questions about its taxonomic classification and inclusion in the *Fervidobacterium* genus. There is not a standardized threshold for the taxonomical genus level, and it has been suggested that this boundary should be estimated for each group independently rather than relying on a universal threshold value [71]. However, our results suggest that the classification of *F. thailandense* within *Fervidobacterium* should be revised and considered to be reclassified in a different genus. All these taxonomic revisions of *Fervidobacterium* clade are also supported by the Genome Taxonomy Database (GTDB), based mainly on genome phylogeny [72–75].

The pan-genome analysis revealed that the core genome was formed by only 5% of the total number of genes, indicating an open pan-genome in the genus *Fervidobacterium* [76], similar to other thermophilic fermentative anaerobes [77] and extremophiles [78]. Additionally, the openness of the *Fervidobacterium* pan-genome was estimated using Heap’s law. The gamma exponent after 1000 iterations was 0.44, which was consistent with an open pan-genome, indicating that its size increases as more genomes are included [79]. Furthermore, a closer inspection of the cloud genes, which were present only in one or two strains, showed that most of them were either transposase-related genes or features of unknown function. Of the 2,709 singletons in the pan-genome, 1,352 were found in *F. thailandense* FC2004^T^, along with 30 horizontally transferred regions detected by Alien Hunter. Pan-genome singletons are usually acquired via horizontal gene transfer [76]. These characteristics of the *F. thailandense* FC2004^T^ genome may be a consequence of dramatic genomic reorganization and recombination, resulting in a very different G + C content and explaining the distant phylogenetic position of this species compared to the other members of the group, and suggesting a possible reclassification to a different genus.

## Proposed reclassifications

The genomic and phylogenomic analyses conducted in this work showed that the following members of genus *Fervidobacterium* should be reclassified (Figure S5 and Figure S6):

*F. riparium* was described as a novel species in 2011 with some minor physiological differences from other *Fervidobacterium* species [15]. In the absence of the genome sequence, experimental DNA-DNA hybridization revealed 20% relatedness with its closest relative, *F. gondwanense* [15]. However, using in silico-based methods, *F. riparium* shares dDDH identity (82.9%) and OrthoANI (98.4%) values well above the species threshold compared to *F. gondwanense* and should thus be members of the same species. This conclusion is supported by GTDB, which considers *F. riparium* as a “false species representative” and includes it in the *F. gondwanense* species cluster (https://gtdb.ecogenomic.org/genome?gid=GCA_025370035.1). We therefore propose to reclassify *F. riparium* as *Fervidobacterium gondwanense* subsp. *riparium* comb. nov.

*F. changbaicum* was described as a novel species in 2007 based on a DNA-DNA hybridization value of 20.5% and different phenotypic features compared to the closest species in the genus, *F. islandicum* [14]. However, our TYGS and OrthoANI results demonstrate that *F. changbaicum* and *F. islandicum* belong to the same genome species with dDDH and OrthoANI values of > 89% and > 98.7%, respectively, also supported by GTDB (https://gtdb.ecogenomic.org/searches?s=al&q=changbaicum). So, based on the earlier valid publication of *F. islandicum*, we propose to reclassify *F. changbaicum* as *Fervidobacterium islandicum* subsp. *changbaicum* comb. nov.

“*F. pennivorans”* DYC [80] should be reclassified as a novel species, *Fervidobacterium ngatamarikiense* sp. nov. (*ngatamarikiensis,* pertaining to the Ngatamariki geothermal area in New Zealand from where the strain was isolated). The type strain is DYC^T^.

*Fervidobacterium* sp. GSH should be renamed as *Fervidobacterium pennivorans* subsp. *shakshaketiis* subsp. nov. (*shakshaketiis*, pertaining to Shakshaketi, the geothermal site in Georgia from which it was isolated).

*Fervidobacterium*. sp. DSM 21710 should be named *Fervidobacterium pennivorans* subsp. *carthaginiensis* subsp. nov. (*carthaginiensis*, pertaining to Carthago, the Roman name for Tunisia, from where it was isolated).

## Supplementary Information

Supplementary Material 1.

Supplementary Material 2.

Supplementary Material 3.

## Data Availability

The complete genome sequences described have been deposited in GenBank and are available under their correspondent accession numbers: F. pennivorans GSH (CP126982), Fervidobacterium sp. 13,770 (CP126498), F. islandicum H-21 T (CP126499), Fervidobacterium sp. 21,710 (CP126500), F. gondwanense DSM13020T (CP126501), and F. thailandense FC2004T (CP140110).
