# Supplementary material for: Comparative genomics of Fervidobacterium: a new phylogenomic landscape of these wide-spread thermophilic anaerobes
Source: BMC Genomics. 2024 Dec 26;25:1248. doi: 10.1186/s12864-024-11128-x (PMC11673897; doi:10.1186/s12864-024-11128-x)
Supplement: Supplementary file 1 — Supplementary Material 1. [file 12864_2024_11128_MOESM1_ESM.docx]

SUPPLEMENTARY MATERIAL

**Comparative Genomics of *Fervidobacterium*. A new phylogenomic landscape of these wide-spread thermophilic anaerobes**

**Rubén Javier-López, Natia Geliashvili and Nils-Kåre Birkeland**

Table S1. Raw and trimming data of the Nanopore and Illumina reads.

|  | *Fervidobacterium* sp.  13770 | *Fervidobacterium* sp.  21710 | *Fervidobacterium* sp.  GSH | *Fervidobacterium gondwanense* DSM13020^T^ | *Fervidobacterium islandicum*  H-21^T^ | *Fervidobacterium thailandense* FC2004^T^ |
| --- | --- | --- | --- | --- | --- | --- |
| **Nanopore** |  |  |  |  |  |  |
| Reads (raw) | 104,770 | 163,423 | 67,335 | 342,936 | 62,845 | 196,973 |
| Reads (trimmed) | 49,956 | 64,089 | 35,090 | 69,117 | 36,331 | 83,086 |
| Bases | 599,207,212 | 521,669,816 | 394,491,279 | 693,624,806 | 496,105,045 | 698,290,295 |
| Coverage (raw) | 280 | 248 | 195 | 319 | 226 | 336 |
| Coverage (after trimming) | 252 | 212 | 176 | 235 | 204 | 303 |
| **Illumina** |  |  |  |  |  |  |
| Reads | 9,954,786 | 8,782,778 | 14,302,014 | 11,088,210 | 15,995,174 | 2,743,526 |
| (Trimmed) | 8,551,410 | 7,719,502 | 13,650,148 | 10,619,904 | 14,942,726 | 2,642,066 |
| Bases | 1,503,172,686 | 1,326,199,478 | 2,159,604,114 | 1,663,231,500 | 2,415,271,274 | 414,272,426 |
| Coverage* | 698 | 627 | 1,060 | 764 | 1,094 | 200 |
| Coverage (after trimming) | 599 | 551 | 1,012 | 732 | 1,022 | 194 |


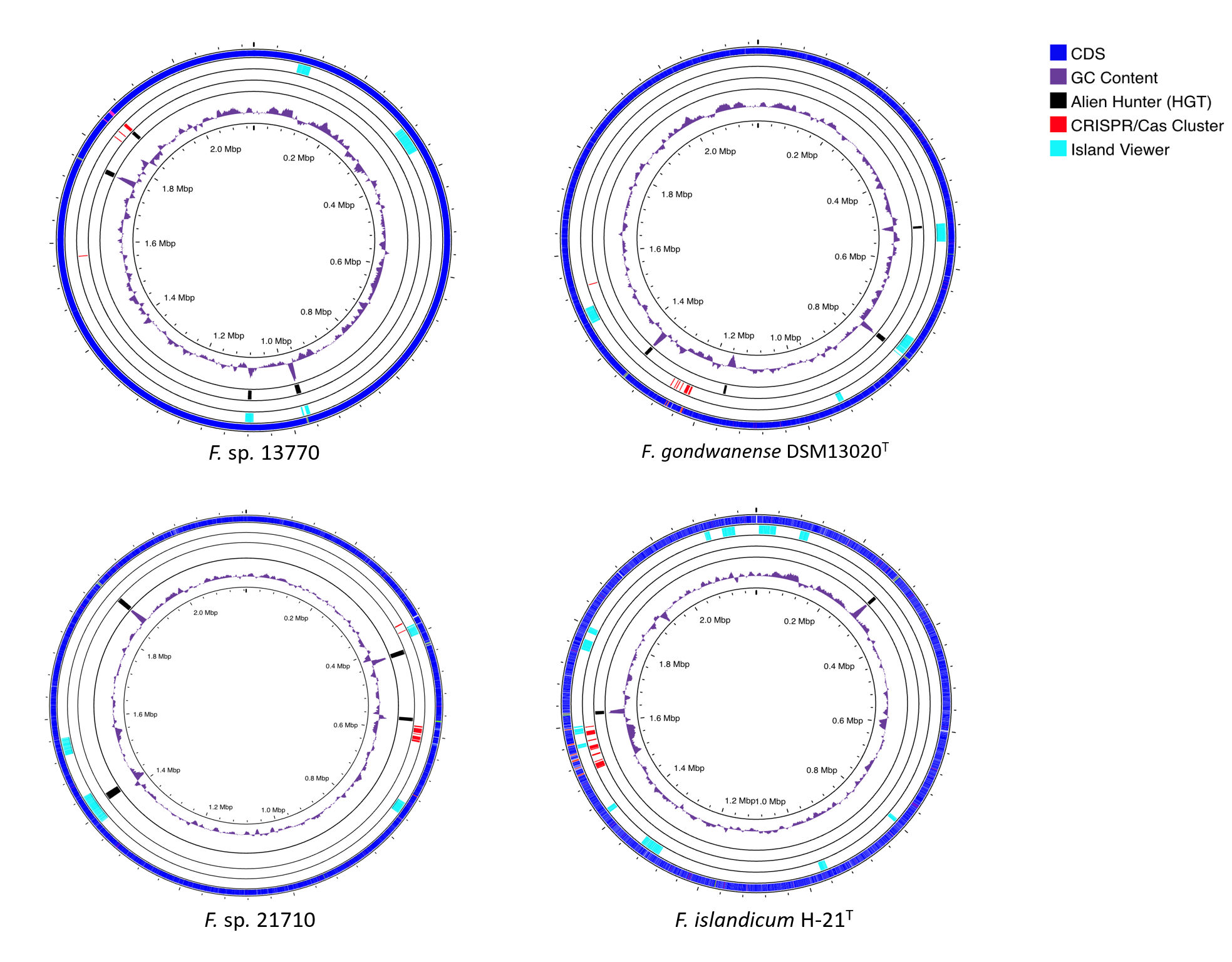


Figure S1. Different features annotated in fervidobacteria described in this study. Each set of rings represents the analyzed genomes. From inner to outermost: backbone (black), G+C content (purple), regions of horizontal gene transfer predicted by Alien Hunter (black), CRISPR/Cas9 clusters found by CRISPR/CasFinder (red), genomic islands annotated with IslandViewer (skyblue), and coding sequences (blue). The figure was made using Proksee.


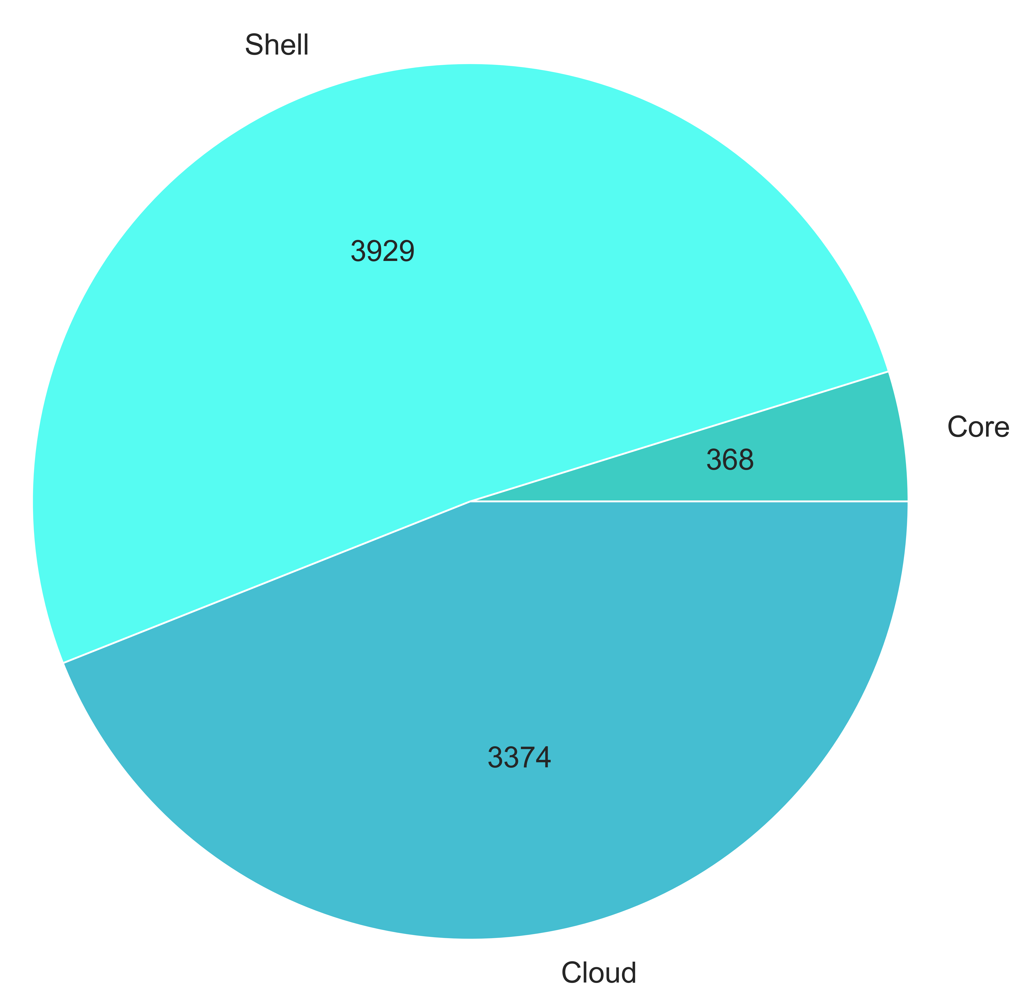


Figure S2. Pie chart representing the *Fervidobacterium* pan-genome. The turquoise portion of the chart corresponds to the core pan-genome, with 368 genes; the light sky-blue portion represents the shell pan-genome comprising 3,829 genes; and the dark sky-blue portion represents the cloud pan-genome composed of 3,374 genes.


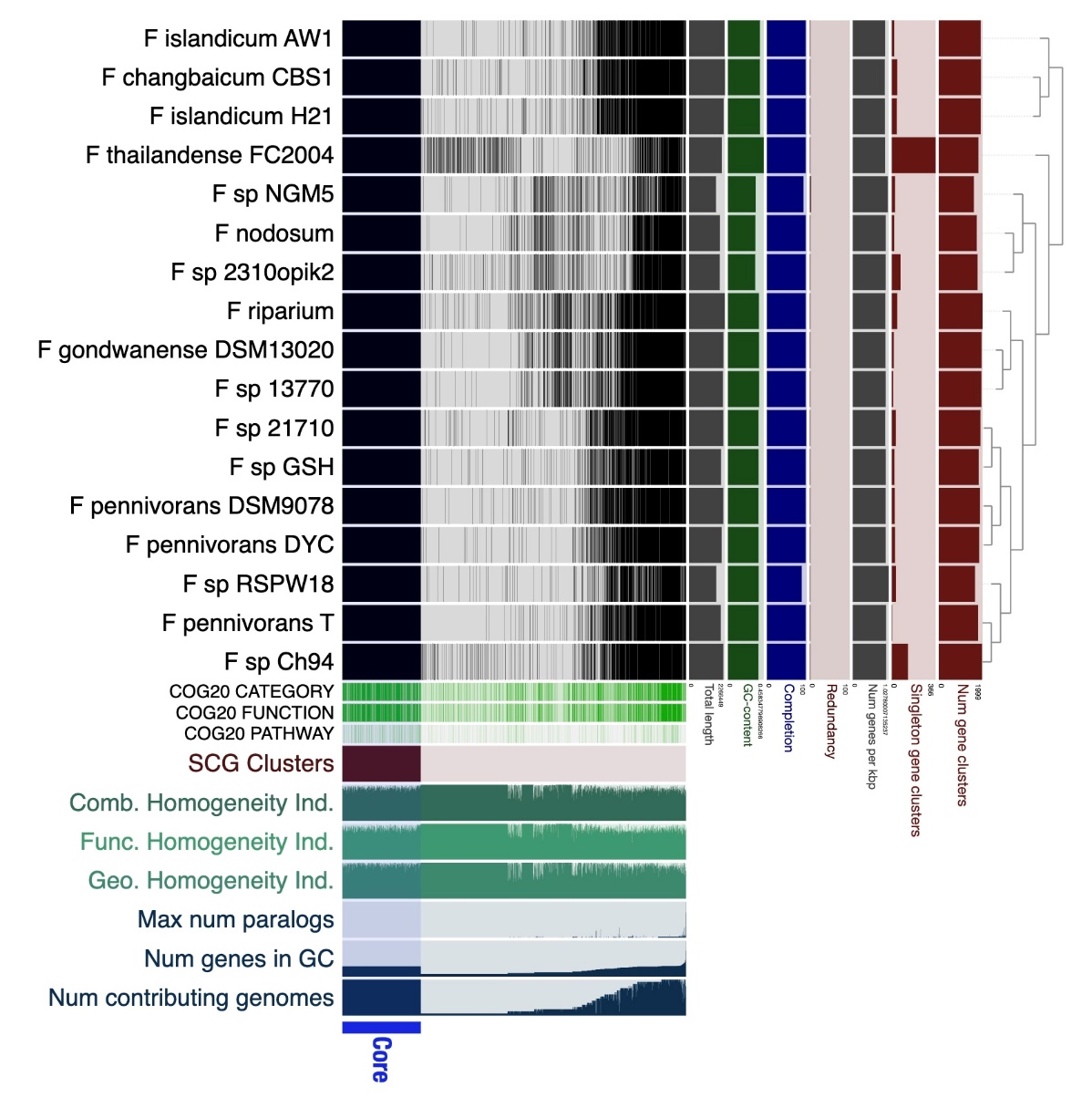


Figure S3. Pan genome matrix and tree constructed with Anvi’o pipeline. The shared fractions (black bars), single copy core genes (SCG) and genes assigned to COG categories are shown.


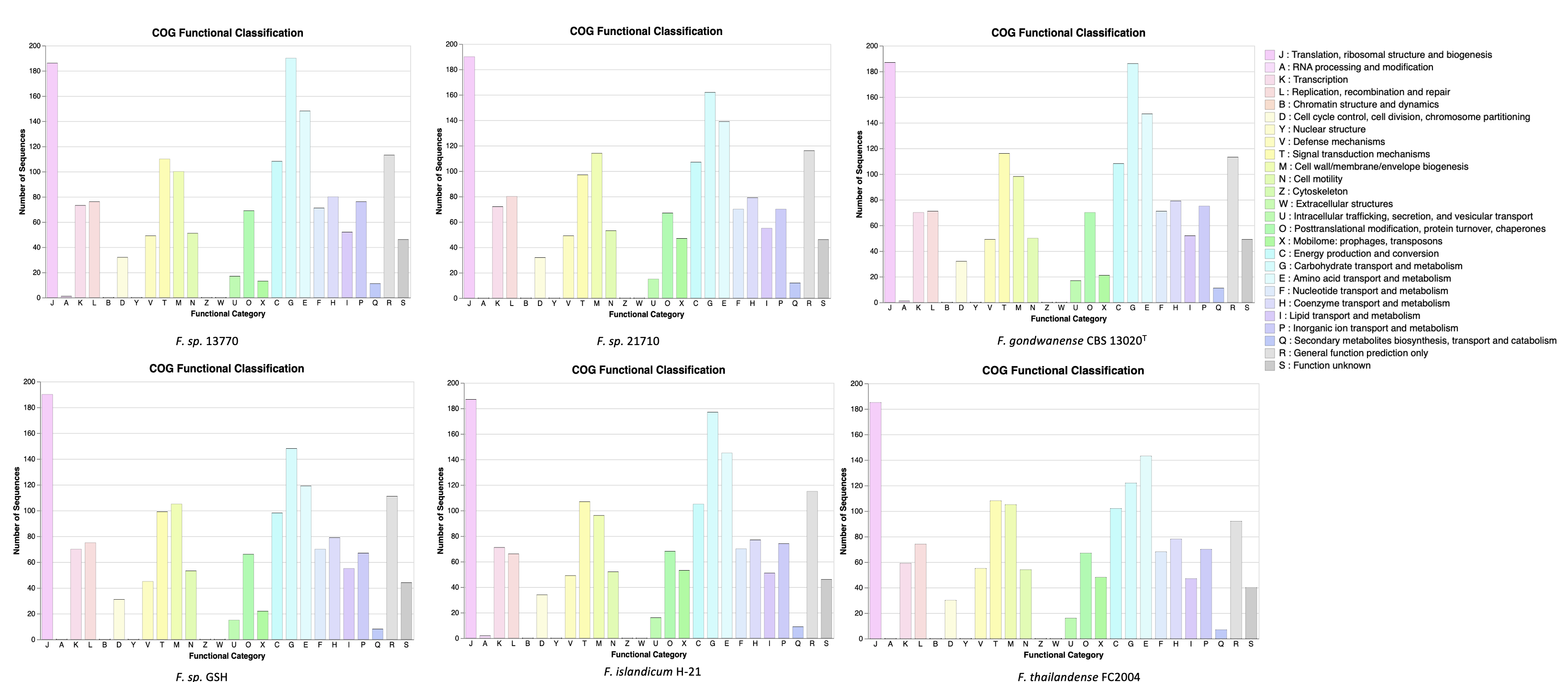


Figure S4. Barchart of Clusters of Orthologous Genes (COG) annotated and classified by COGClassifier in the genomes described in this work. The x axis of each sample shows the different functional categories and the y axis the number of genes found.


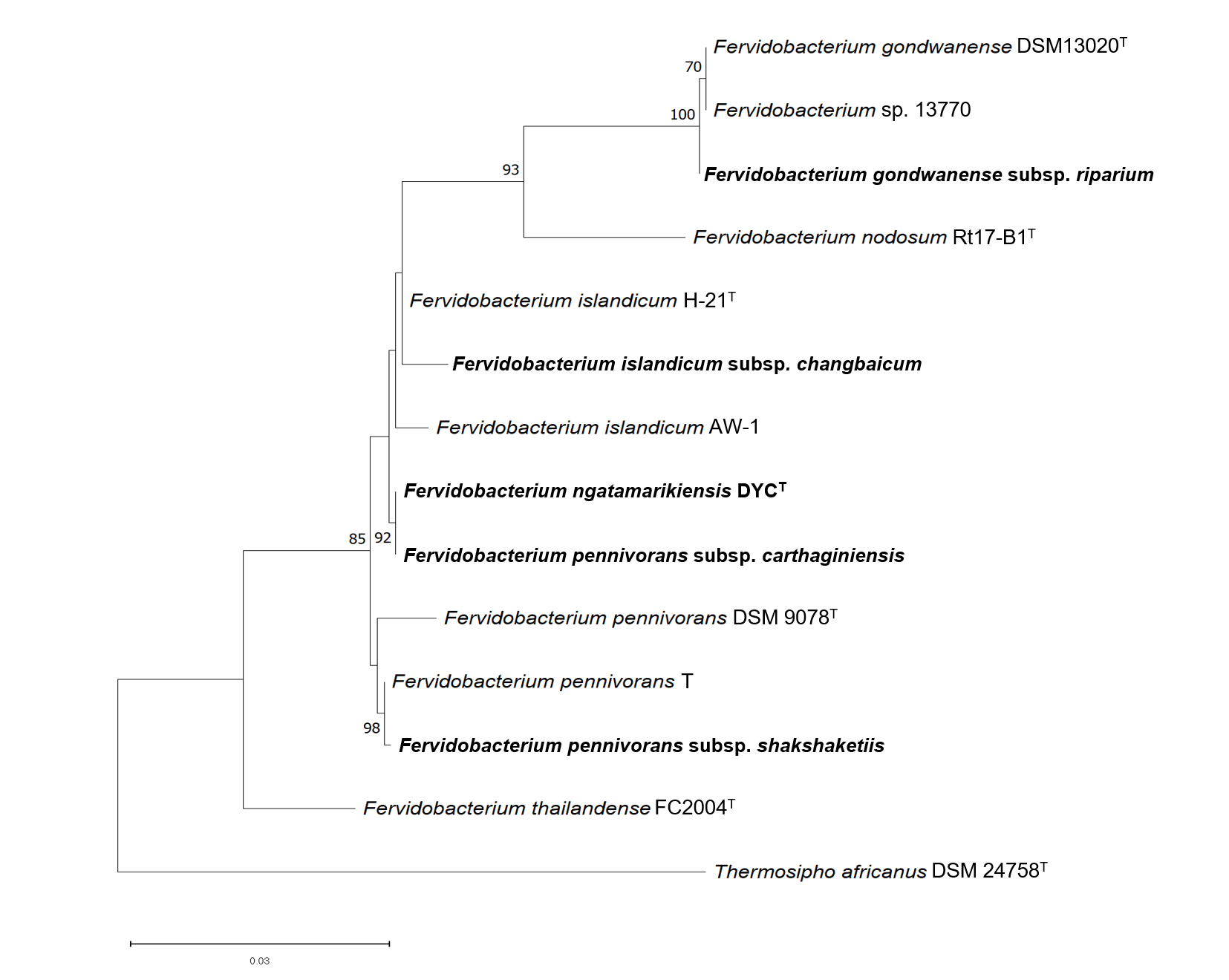


Figure S5. Maximum likelihood phylogenetic tree showing the evolutionary history of the genus *Fervidobacterium* based on the 16S rRNA gene, as shown in Figure 3. The suggested new names for *F. riparium*, *F. changbaicum*, *F. pennivorans* DYC, *Fervidobacterium* sp. 21710 and *Fervidobacterium* sp. GSH are updated and highlighted in bold letters.


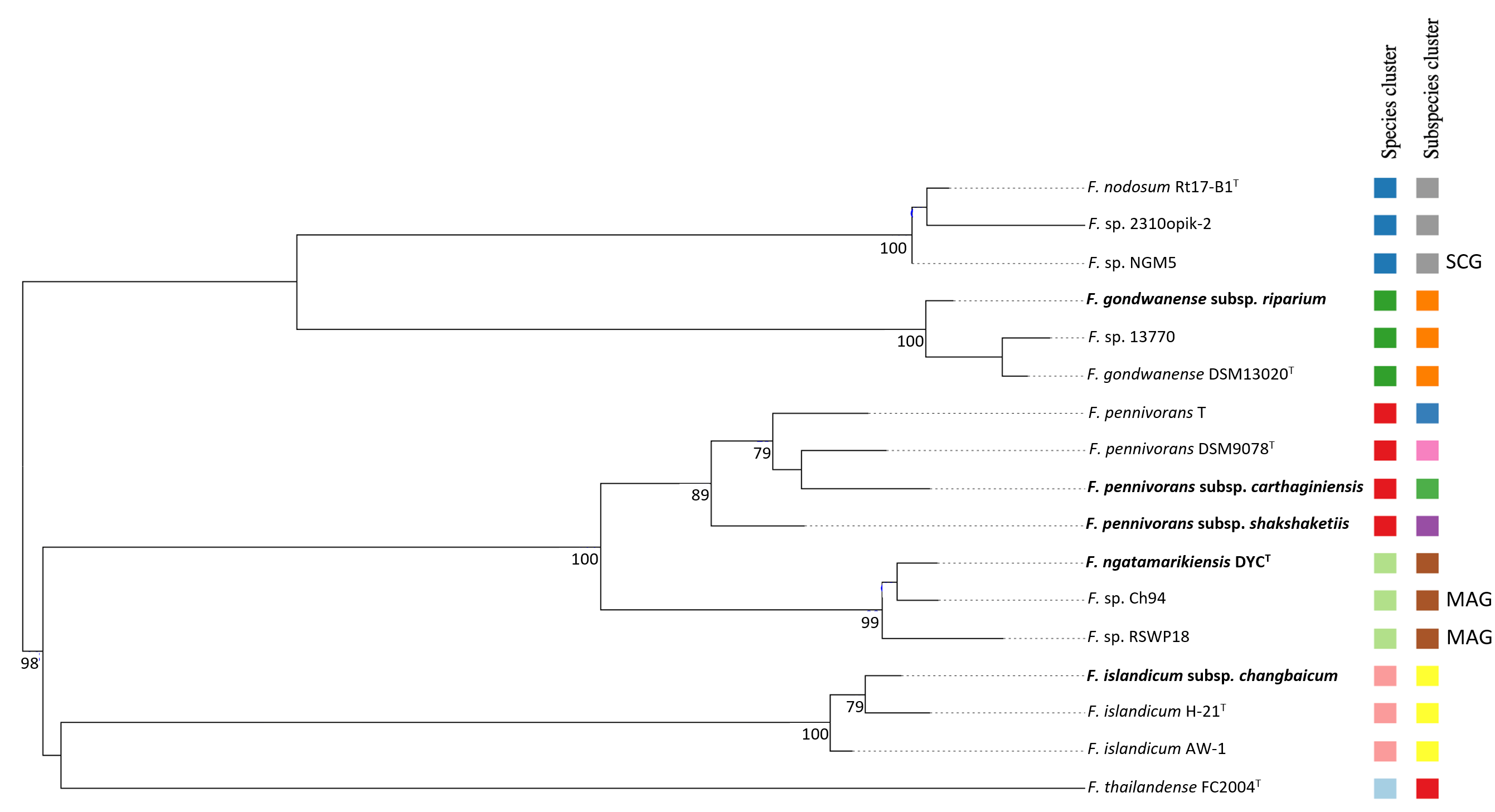


Figure S6. Phylogenomic tree of the *Fervidobacterium* species, strains, metagenome-assembled genomes (MAGs), and single-cell genome (SCG) constructed using the TYGS genome server, as shown in Figure 4. The suggested new names for *F. riparium*, *F. changbaicum*, *F. pennivorans* DYC, *Fervidobacterium* sp. 21710 and *Fervidobacterium* sp. GSH are updated and highlighted in bold letters.
